# Revealing the Molecular Interactions between Human ACE2 and the Receptor Binding Domain of the SARS-CoV-2 Wild-Type, Alpha and Delta Variants

**DOI:** 10.3390/ijms24032517

**Published:** 2023-01-28

**Authors:** Cécilia Hognon, Emmanuelle Bignon, Antonio Monari, Marco Marazzi, Cristina Garcia-Iriepa

**Affiliations:** 1 Departamento de Química Analítica, Química Física e Ingeniería Química, Universidad de Alcalá, Ctra. Madrid-Barcelona, Km 33,600, 28871 Alcalá de Henares, Madrid, Spain; 2 UMR 7019 LPCT, Université de Lorraine and CNRS, F-5400 Nancy, France; 3 ITODYS, Université Paris Cité and CNRS, F-75006 Paris, France; 4 Instituto de Investigación Química “Andrés M. del Río” (IQAR), Universidad de Alcalá, 28871 Alcalá de Henares, Madrid, Spain

**Keywords:** SARS-CoV-2, Alpha variant, Delta variant, molecular dynamics, ACE2/RBD complex formation, protein-protein interactions

## Abstract

After a sudden and first spread of the pandemic caused by the novel SARS-CoV-2 (Severe Acute Respiratory Syndrome—Coronavirus 2) wild-type strain, mutants have emerged which have been associated with increased infectivity, inducing surges in the contagions. The first of the so-called variants of concerns, was firstly isolated in the United Kingdom and later renamed Alpha variant. Afterwards, in the middle of 2021, a new variant appeared called Delta. The latter is characterized by the presence of point mutations in the Spike protein of SARS-CoV-2, especially in the Receptor Binding Domain (RBD). When in its active conformation, the RBD can interact with the human receptor Angiotensin-Converting Enzyme 2 (ACE2) to allow the entry of the virions into cells. In this contribution, by using extended all-atom molecular dynamic simulations, complemented with machine learning post-processing, we analyze the changes in the molecular interaction network induced by these different strains in comparison with the wild-type. On one hand, although relevant variations are evidenced, only limited changes in the global stability indicators and in the flexibility profiles have been observed. On the other hand, key differences were obtained by tracking hydrophilic and hydrophobic molecular interactions, concerning both positioning at the ACE2/RBD interface and formation/disruption dynamic behavior.

## 1. Introduction

Mutations in viral genes are the main strategy for viruses to increase their virulence and dissemination. This way, they can elude the available therapeutic strategies and/or the immune system, in some cases, causing changes in the symptoms or severity of the disease induced by the virus [1]. Coronaviruses (CoV) are a large family of RNA viruses, whose mutation rates are higher than those of DNA viruses [2,3]. This family includes SARS-CoV-2, responsible of Severe Acute Respiratory Syndrome (SARS) and the related COVID-19 disease [4]. Since the beginning of the pandemic, in the late 2019, different variants have emerged [5,6]. The first one appeared in September 2020, where the sudden surge of COVID-19 cases in the United Kingdom (UK) was associated to the development of a novel variant, originally styled UK variant and later renamed Alpha variant. The Alpha variant is the first of the so-called Variants Of Concerns (VOC) that has been identified by the World Health Organization (WHO), and rapidly became the dominant strain not only in the UK but also in most of the world [7]. Despite the morbidity of the Alpha variant is similar to the one of the wild-type (WT), it is outstanding its increased infectivity, which has been estimated around 50% higher than for the WT [8]. Afterwards, the Delta variant emerged in the middle of 2021 and quickly became the world dominant strain characterized by a ca. 60% higher transmissibility compared to the Alpha variant, even despite vaccination [9]. SARS-CoV-2 continues to evolve and adapt to the human population but, interestingly, the number of variants found is relatively small compared to other RNA viruses. This is due to the presence of an exonuclease that repairs the errors and point mutations occurring during RNA replication [10].

SARS-CoV-2 is an enveloped virus whose external lipid membrane shows the presence of structural proteins including, most notably, the Spike protein, that protrudes from the membrane giving the characteristic crown (in Latin: Corona) shape to the virus. The Spike protein is fundamental to initiate the viral cycle, since it is responsible for binding with the human receptors, mainly the Angiotensin-Converting Enzyme 2 (ACE2), thus promoting the fusion of viral and cellular membranes and hence, the entrance of the virus into the cell [11,12,13,14].

**Figure 1:**
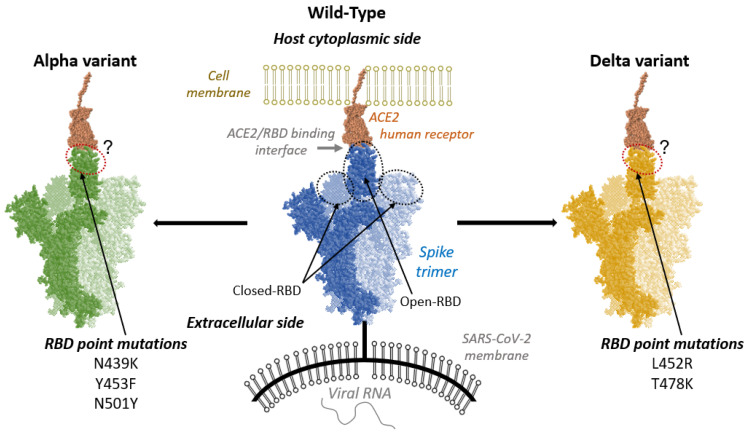
Schematic representation of the interaction between ACE2 receptor (brown) and Spike-RBD (blue: wild-type; green: Alpha variant; orange: Delta variant). In each case, the two light-colored Spike monomers have a RBD in closed conformation, while the dark-colored Spike monomer has a RBD in its open conformation, allowing complexation with ACE2. For both variants the RBD point mutations are listed.

From a structural and biophysical point of view, the Spike protein is a glycoprotein organized as a transmembrane homotrimer. The Spike protein is composed of different units, among which the Receptor Binding Domain (RBD) is of crucial importance since, in its active conformation, it is capable of forming a complex with the human ACE2 receptor, hence triggering the infection (Figure 1) [15,16]. For this reason, the spike protein is one of the main targets for the design of anti-viral drugs and especially vaccines [17,18,19,20,21]. As a matter of fact, the structure of the spike protein, as well as of the complex between RBD and ACE2, has been the subject of various experimental and computational works aimed not only at identifying the interaction patterns, but also the subtle conformational equilibrium of the Spike protein itself. For instance, it has been shown that, compared to SARS-CoV, SARS-CoV-2 favors a closed (inactive) conformation of RBD while leading to stronger interactions with ACE2 [22] (Figure 1). In addition, the thermodynamics of the binding between ACE2 and RBD have also been investigated thanks to experimental measurements and more recently, by enhanced sampling molecular dynamics (MD) simulations [23,24,25]. According to experimental data, wild-type and Alpha strains have a similar binding affinity (11.4 [26] and 11.7 [27] kcal/mol respectively) while Delta variant exhibits a reduced affinity (9.9 kcal/mol) [28]. The role of the glycan shield in shaping conformation flexibility and binding capacity of the Spike protein has also been investigated using both all-atom and coarse-grain MD simulation approaches [29,30]. The ACE2 binding region is formed by a rather extended α-helix, which is in direct contact with the RBD through a wide hydrogen bonding network, also including the participation of interfacial water molecules [24,31].

Among the point mutations present in the Alpha and Delta variants, some of them are located in the RBD, and hence can have a specific effect in tuning the binding affinity with ACE2, thus influencing the virulence of the mutated strain. The point mutations included in the RBD of the Alpha variant correspond to N501Y, Y453F, and N439K (Figure 1). The first one is usually pinpointed as the most important factor in increasing the virus aggressiveness, as it is located in the middle of the RBD/ACE2 binding area [32,33].

Concerning the Delta variant, it presents two point mutations located in the RBD region: L452R and T478K (Figure 1). It was proposed that these two mutations induce significant perturbations in van der Waals and electrostatic interactions [34].

Moreover, independently on the variant, point mutations can affect not only the ACE2-RBD interactions but also the equilibrium between active (open) and inactive (closed) RBD conformations, or even facilitate the elusion of the immune system by reducing the exposed viral protein surface [35,36,37,38]. Indeed, the L452R mutation of the Delta variant is associated with immune system escape, resulting in a transmissibility improvement [35,39,40].

In the present contribution, we aim to discern at the atomistic level the characteristic features of the ACE2-RBD interaction for the Alpha and Delta variants and compare them to the ones found for the original wild-type (from now on, WT) system. Especially, and differently from previously presented investigations, we will pay attention to the effects of the point mutations on the modification of hydrophilic and hydrophobic interactions, relating them to the rigidity of the ACE2-RBD interface evaluated through a machine learning-based approach.

This study was performed by modeling the active RBD in interaction with ACE2, without explicitly including the full Spike protein nor its glycosylated sites. Indeed, as previously shown by us and others, the rest of the Spike protein does not take part (not even indirectly) in the formation and stabilization of the ACE2-RBD interface [17,26], and no one glycosylated site is located at the RBD surface [41,42] that is in contact with ACE2.

## 2. Results and Discussion

In this section, we investigate from a molecular point of view, the impact of the point mutations found in the RBD of Alpha and Delta variants, compared to the WT system. Especially, we analyze in detail the variations of the full ACE2/RBD interaction region, accumulating insights into how local modifications could induce changes in the secondary and eventually tertiary structures of the RBD, thus possibly influencing the overall ACE2-RBD interaction and rigidity. In the following, we present and discuss the results obtained regarding the dynamic behavior, hydrophilic and hydrophobic ACE2-RBD interaction network, and structural flexibility of the three systems under study: ACE2 in interaction with WT-RBD, Alpha-RBD, and Delta-RBD.

### 2.1. Overall Structural Analysis

Three independent MD simulations of 500 ns each have been performed for each molecular system, i.e., ACE2/WT-RBD, ACE2/Alpha-RBD and ACE2/Delta-RBD.

The Root Mean Square Deviation (RMSD) of the respective backbones was evaluated (i.e., ACE2 and RBD protein backbones), highlighting the stability of the different complexes. In particular, the average RMSD values of the complexes are 3.4 Å, 3.8 Å and 4.2 Å, for WT, Alpha and Delta variants, respectively (Table 1 and Figure S1). Indeed, the complexes’ stability is confirmed by both ACE2 and RBD backbone stability (Table 1 and Figures S2 and S3).

Furthermore, we have analyzed the stability of the ACE2/RBD interaction for the three studied systems measuring the distance between the centers of mass of ACE2 and RBD along the simulation time: the limited fluctuations of this distance (ca. 4 Å) indicate in each case a notable stability of the ACE2/RBD complex (Figure S4). Concerning the average distance, no significant differences (i.e., less than 1 Å; see Table 1) are found. This finding confirms that the differences in binding energy between ACE2 and RBD (almost negligible for WT and Alpha variant—11.4 [26] and 11.7 [27] kcal/mol, respectively—but lower for the Delta variant—9.9 kcal/mol [28]) are not simply due to an overall closer/wider arrangement between the two sub-units. Therefore, a more sophisticated analysis is required, as it will be detailed in the following sections.

### 2.2. Analysis of the Molecular Interactions

With the aim of rationalizing the mutation effect on the ACE2/RBD interaction strength, in this section we will discuss both, the hydrophilic (hydrogen bonds and eventually salt bridges) and hydrophobic interactions found along the simulations performed for the three systems under study (i.e., ACE2/WT-RBD, ACE2/Alpha-RBD and ACE2/Delta-RBD). Furthermore, the Solvent Accessible Surface Area (SASA) computed for the three systems is compared and the trends found rationalized in light of the type of interaction.

**Figure 2:**
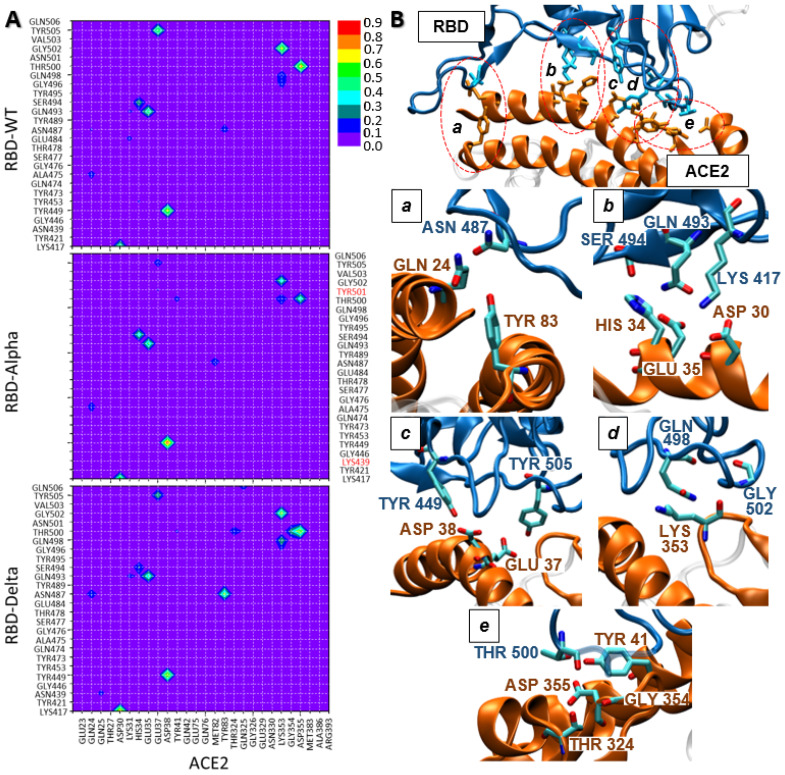
(**A**) 2D maps of hydrophilic interactions between WT-, Alpha-, and Delta-RBD and ACE2 residues. (**B**) Representative snapshots showing the amino acids involved in the main hydrophilic interactions. Snapshots are extracted from the structure ACE2/WT-RBD cluster 0. Sub-figures (**a**–**e**) correspond to different fractions of the ACE2/RBD interaction region.

Starting with the hydrophilic interactions, diverse hydrogen bonds have been identified for the ACE2/WT-RBD reference system. It should be stressed that hydrogen bonds extended all over the ACE2/RBD interaction region (see Figure 2A), suggesting an important role to promote the ACE2/RBD interaction, as previously found [17,22]. Four of these hydrogen bonds are formed in more than 55% of the simulation time: Thr500-Asp355, Gly502-Lys353, Tyr449-Asp38, Tyr505-Glu37 (Figure 2A and Table S1). Whereas, other three weaker hydrogen bonds have been found in 30–48% of the simulation time: Lys417-Asp30, Gln493-Glu35, Ser494-His34 (Figure 2A and Table S1). It should be remarked that Lys417 and Asp30 form a salt bridge, strengthening the interaction. Some of these hydrogen bonds have been already reported for the ACE2/WT-RBD interaction [23,26,43].

To identify the eventual probability of having all these interactions simultaneously, we have analyzed the average structure of the three most representative ACE2/WT-RBD clusters (see Table S3): cluster 0 is characterized by these seven hydrogen bond interactions, leading to a rather strong ACE2/WT-RBD interaction. However, cluster 1 shows a weaker hydrophilic interaction pattern, with only two of these hydrogen bonds whereas, cluster 2 presents an intermediate behavior showing five of them. Therefore, for ACE2/WT-RBD we can conclude that hydrophilic interactions play an important role, possibly leading to an extended hydrogen bonding network, as found for cluster 0.

The analysis of the hydrogen bonding patterns found for the ACE2/Alpha-RBD system shows clear differences with respect to the ACE2/WT-RBD reference (Figure 2A and Table S1): first, the intensity of three hydrogen bonds has been increased: Tyr449-Asp38 (15% increase), Ser494-His34 and Lys417-Asp30 (both 10% increase). However, three of the ACE2/WT-RBD hydrogen bonds are weakened: (i) Tyr505-Glu37 interaction is quite weak (37% decrease); (ii) Gly502-Lys353 (0.28% decrease) and (iii) Thr500-Asp355 is weakened but its partial loss is compensated by the formation of Thr500-Tyr41 and Thr500-Lys353, leading to an overall similar intensity (*ca.* 76%) compared to ACE2/WT-RBD. Hence, we can conclude that, overall, the nature and strength of hydrogen bond interactions in ACE2/WT-RBD and ACE2/Alpha-RBD are similar, leading to just a slight weakening of these interactions for the Alpha variant. Also, it should be mentioned that none of the Alpha-RBD point mutation is directly involved in any hydrogen bond with ACE2.

Regarding the ACE2/Delta-RBD system, some outstanding differences arise compared to ACE2/WT-RBD. The most evident one is the strong Asn487-Tyr83 hydrogen bond interaction, which is much weaker in the ACE2/WT-RBD and ACE2/Alpha-RBD complexes (Figure 2A and Table S1). It should be stressed that the Asn487 residue also interacts with Gln24 (*ca.* 20%), leading to an overall rather strong hydrogen bonding interaction. Moreover, the Lys417-Asp30 and Gln498-Lys353 interactions are stronger in ACE2/Delta-RBD compared to the other two systems (*ca.* 20% and 10% increase, respectively). Regarding the weakening of the hydrogen bond interactions, compared to the WT system, the decrease of the strength of Tyr505-Glu37 (*ca.* 25%) is evident. Remarkably, this interaction was even weaker in the ACE2/Alpha-RBD complex. In addition, two more hydrogen bonds are weakened, yet to a lesser extent: Gly502-Lys353 (20%) and Thr500-Asp355 (25%). However, while Thr500-Asp355 interaction is weakened, it is compensated by the formation of additional hydrogen bonds between Thr500 and other ACE2 residues such as Gly354 (24%), Thr324 (17%) and Tyr41 (11%), leading to an overall consistent hydrogen bond interaction of Thr500 during all the simulation time (100%). This interaction appears even stronger than for the other two systems under study. Hence, we can conclude that the strong Asn487-Tyr83 hydrogen bond interaction is a characteristic of the ACE2/Delta-RBD system although this residue has not been mutated in the Delta variant. However, it is evident that the two point mutations located in the Delta-RBD region should involve a change in the spatial organization of the RBD residues, leading to a different hydrophilic pattern. This is a distinctive feature of ACE2/Delta-RBD, while the ACE2/Alpha-RBD hydrophilic pattern is quite similar to the one of ACE2/WT-RBD.

**Figure 3:**
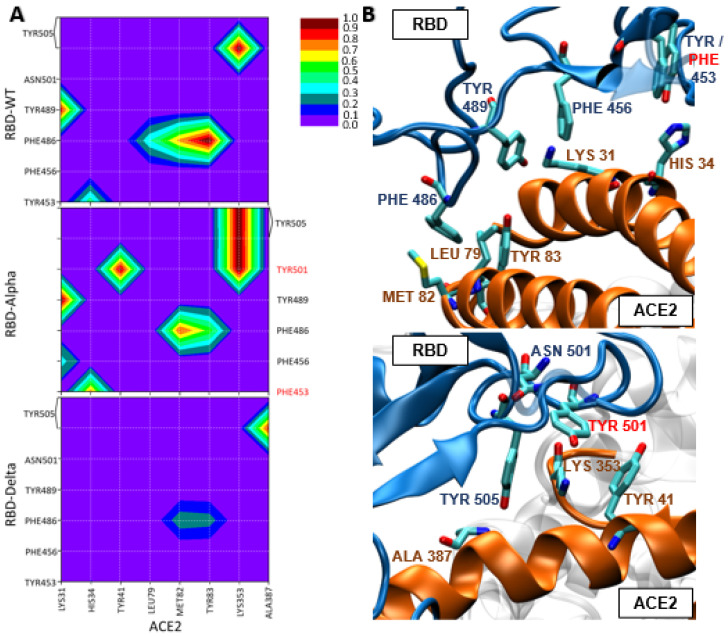
(**A**) 2D maps of hydrophobic interactions between WT, Alpha or Delta-RBD and ACE2 residues. (**B**) Representative snapshots showing the amino acids involved in the hydrophobic interactions. Snapshots are based on the structures of ACE2/Alpha-RBD cluster 0 and ACE2/WT-RBD cluster 2. Point mutations are depicted in red.

Once we have rationalized the hydrophilic interactions and patterns for the three systems under study, we will examine the role of hydrophobic interactions in the ACE2/RBD interface region. Again, we firstly analyze the interactions found for the ACE2/WT-RBD reference system. By observing Figure 3A (and Table S2), we can conclude that relevant hydrophobic interactions are present along the whole ACE2/RBD interaction region (Figure 3B). In particular, four WT-RBD residues lead to quite strong hydrophobic interactions with ACE2: (i) Tyr505 offers a π-alkyl interaction with Lys353, kept along all the simulation time; (ii) Tyr489 forms a π-alkyl interaction with Lys31 in ca. 90% of the simulation time; (iii) Phe486 leads to hydrophobic interactions with two near ACE2 residues, namely Met82 (π-alkyl interaction during 72% of the simulation time) and Tyr83 (π-π T shaped and π-π stacked interactions during 49% and 51% of the simulation time, respectively); (iv) Tyr453 is involved in a π-π T shaped interaction with His34 during 46% of the simulation time. At this point, the key role of residue Tyr505 should be remarked, as it is involved in both strong hydrophilic and hydrophobic interactions. Hence, we can conclude that the stable and strong interaction found between ACE2 and WT-RBD could be due to the combination of hydrophilic and hydrophobic interactions along the whole ACE2/WT-RBD contact region.

Afterwards, we evaluate the possible effect of the RBD point mutations on the hydrophobic interaction pattern. First, we compare the hydrophobic interactions found for ACE2/Alpha-RBD with the already analyzed ACE2/WT-RBD ones. Observing Figure 3A (and Table S2) we can note some differences among these two patterns. At a first glance we notice that novel and stronger hydrophobic interactions are present in the ACE2/Alpha-RBD interface region compared to the WT system, involving Tyr501, Tyr505, and Phe456. On one hand, Tyr501 is one of the residues mutated for the Alpha variant (N501Y), so the RBD mutation is directly contributing to a stronger ACE2/Alpha-RBD hydrophobic interaction. In particular, this residue interacts both with Lys353, through a π-alkyl interaction (during all the simulation time), and with Tyr41, through a π-π T shaped interaction (during a 94% of the simulation time). On the other hand, Tyr505 and Phe456, although preserved from WT, lead to novel hydrophobic interactions: Tyr505-Lys353 through amide-π staked interaction (during all the simulation time) and partially Phe456-Lys31 π-alkyl interaction (found in the 37% of the simulation time). Apart from novel interactions, we can also observe the strengthening of an already discussed ACE2/WT-RBD hydrophobic interaction, involving residue number 453. This residue is mutated in the Alpha-RBD variant (Y453F), however in this case this mutation keeps the nature of the interaction (π-π T shaped with His34) but increases its strength (*ca.* 30%). Other two hydrophobic ACE2/WT-RBD interactions are maintained in strength and nature (Tyr489-Lys31 and Tyr505-Lys353), whereas the interactions formed with Phe486 are weakened as the π-π stacked Phe486-Tyr83 arrangement is not observed along the ACE2/Alpha-RBD simulation. Hence, we can conclude that the hydrophobic interactions are notably increased for the ACE2/Alpha-RBD system. The mutated residues N501Y and Y453F appear as the key factors in strengthening the ACE2/RBD interaction. These residues could compensate the similar but weaker hydrophilic pattern found for ACE2/Alpha-RBD compared to the WT complex. Moreover, the relevance of residue Tyr505 is even increased in this system as it is involved in diverse and strong hydrogen bonds and hydrophobic interactions.

Finally, comparing the hydrophobic pattern found for ACE2/Delta-RBD with the other two systems (Figure 3A), we can see that the hydrophobic interactions are clearly and significantly weaker (Table S2). In fact, only two relevant hydrophobic interactions have been found along the ACE2/Delta-RBD simulations: one is Tyr505-Ala387 exerted through π-alkyl interaction (found in 85% of the simulation time). The latter interaction is clearly characteristic of this system as it was not observed in ACE2/WT-RBD and ACE2/Alpha-RBD complexes (where Tyr505 interacts with Lys353). A second hydrophobic interaction involves Phe486, already found in the ACE2/Alpha and WT-RBD complexes, but in this case, it is significantly weakened. Indeed, we may identify the interaction of Phe486-Met82 through π-alkyl interaction and Phe486-Tyr83 through π-π stacked interaction (found in 28% and 25% of the simulation time, respectively). Hence, we can confidently conclude that hydrophobic interactions should play a lesser role in the ACE2/Delta-RBD complex stabilization. This result is in line with the lower affinity measured [28] between ACE2 and Delta-RBD, compared to WT- and Alpha-RBD, as the hydrophilic network found for ACE2/Delta-RBD is not strong enough to compensate this loss of hydrophobic interactions in the ACE2/RBD interface. More in general, this also means that a comprehensive understanding of large-area and non-specific protein-protein interactions, as it is the case here, cannot be obtained by solely evaluating hydrophilic interactions, as usually performed for classical MD analysis, since hydrophobic interactions could play a relevant role.

**Figure 4:**
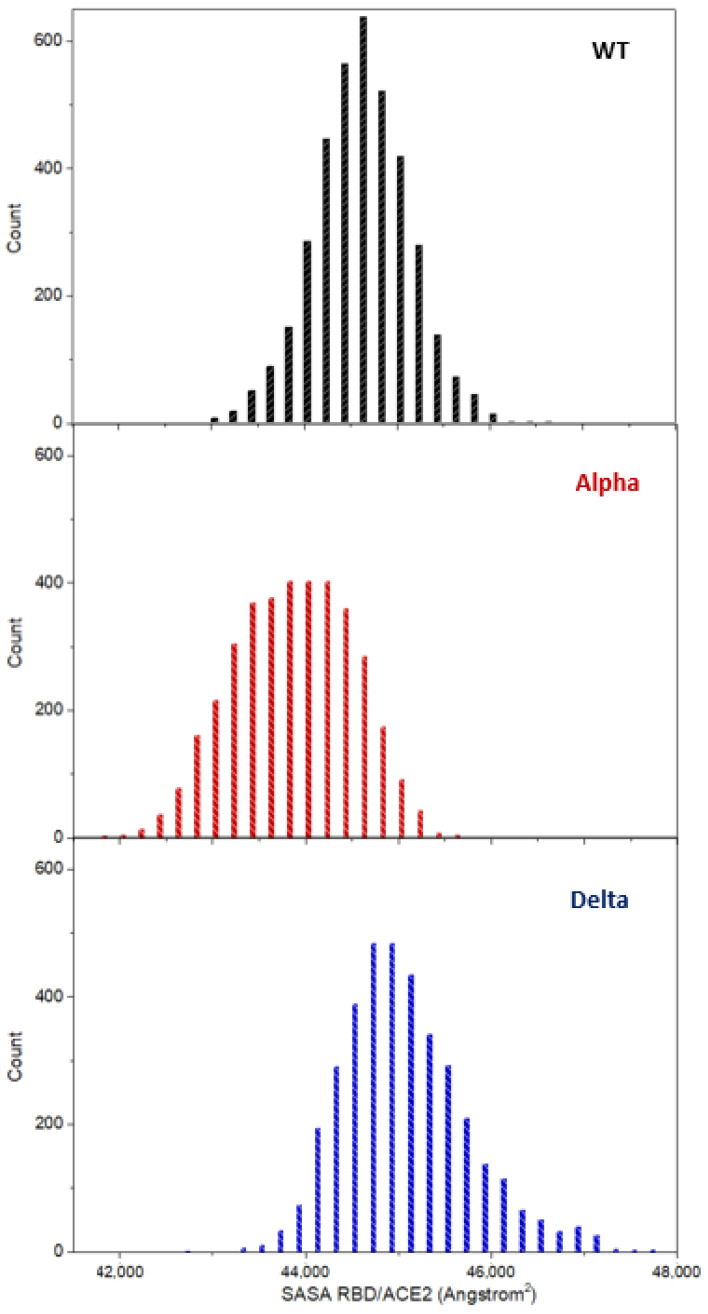
Histogram of the SASA values computed along the simulation time. The data of the three runs of each ACE2/RBD complex under study has been considered to build each histogram.

As an attempt to rationalize the combined effect (including both hydrophobic and hydrophilic interactions) on the ACE2/RBD interface, we have computed the Solvent Accessible Surface Area (SASA) for the three systems under study (Figure 4). It is observed that the SASA computed for the ACE2/Delta-RBD system corresponds to the average largest area and the distribution is the most disperse, spanning almost 5000 Å^2^ (from ca. 43,000 to 48,000 Å^2^). As a reminder, ACE2/Alpha-RBD presents a similar hydrogen bonding pattern with strengthened hydrophobic interactions compared to ACE2/WT-RBD. However, the opposite is observed for ACE2/Delta-RBD, that is, hydrogen bond interactions are slightly increased while hydrophobic interactions are decreased compared to ACE2/WT-RBD. Hydrogen bond interactions at solvent exposed interface may be deemed more flexible than hydrophobic interactions, as they can be weakened and eventually disrupted by water molecules. Hence, the larger the number of hydrogen bond interactions, the more dynamic is expected the formation/disruption equilibrium concerning the ACE2/RBD interface region. Similarly, a larger number of hydrophobic interactions gives a stiffer interaction region, and a more important solvent excluded surface. The more flexible and dynamic the interaction region (corroborated, at the molecular level, by higher hydrophilic interactions and lower hydrophobic interactions), the larger is expected to be the SASA as the accessibility of the solvent (water molecules) is facilitated. On the contrary, the access of the solvent molecules will be prevented in stiffer interaction regions (larger hydrophobic interactions), also due to their non-polar chemical nature with respect to water intrinsic polarity. This is supported by our computed SASAs: the lowest SASA values are obtained for ACE2/Alpha-RBD which presents the more significant and larger hydrophobic interactions, while the largest values are shown for ACE2/Delta-RBD, for which the hydrophobic interactions are much less relevant.

### 2.3. Structural Flexibility

**Figure 5:**
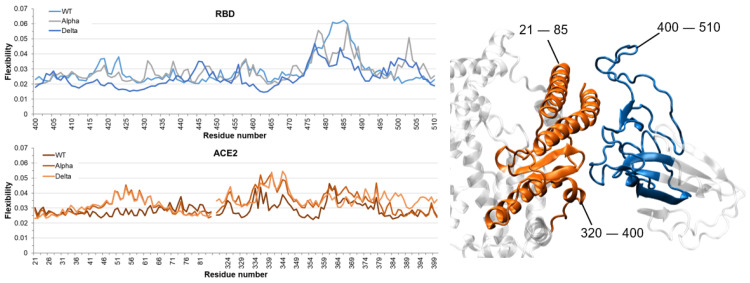
Flexibility profiles of RBD (top) and ACE2 (bottom) residues at the contact surface, computed by machine learning post-analysis, for both Alpha and Delta variants and WT systems. A cartoon representation of the 400–510 residues’ region of RBD (in light and dark blues) and of the 21–85 and 320–400 residues’ regions of ACE2 (in brownish orange) is depicted on the right. This residues’ selection is based on the effective contact region between RBD and ACE2. The full flexibility profiles are shown in Figure S6.

Finally, with the aim of giving more insightful details into the dynamic behavior of the ACE2/RBD interface formation and relative stability, we have evaluated the role of the Alpha-RBD and Delta-RBD point mutations on the flexibility of the corresponding ACE2/RBD complexes. For this aim, the flexibility profiles of ACE2 and RBD (independently) of each complex have been generated by machine learning post-analysis (see Section 3.4 for details), revealing localized perturbations at the ACE2/RBD interface (Figure 5). More precisely, comparing the RBD profiles obtained for WT- and Alpha-RBD, one observes that the general profiles of the flexibility are quite similar, except for a stiffening of the 477–489 residues’ segment and a pronounced increase of the flexibility of the 498–508 residues’ region, where the N501Y mutation is present. Concerning the RBD flexibility of the Delta variant, compared to WT, an overall decrease in protein flexibility has been found along all the RBD sequence, except for 442–447 and 495–505 residues’ regions. Comparing the RBD flexibility profiles of Alpha and Delta variants, we find two similar behaviors: (i) in the 481–485 residues’ region, the WT-RBD flexibility is consistently larger than the Alpha-RBD and especially of the Delta-RBD and, (ii) in the 498–505 residues’ region, where the RBD is more flexible for the two variants compared to WT, with the Alpha variant significantly more flexible, probably due to its N501Y point mutation.

Regarding the ACE2, residues located at the interface with RBD reveal higher flexibility for both Alpha and Delta variants (in the 40–69 and 338–360 residues’ regions). For the Delta variant, the 383–398 residues’ region also shows an increased flexibility.

When overall comparing RBD and ACE2, on one hand, the WT-RBD is more flexible than ACE2, thus possibly facilitating the viral RBD to optimally adapt to human receptors. On the other hand, in ACE2/Delta-RBD, the human ACE2 is more flexible than RBD. Finally, for the Alpha variant, both ACE2 and RBD are similarly flexible, pointing toward a cooperative reshaping of the interacting ACE2 and RBD interfaces. These differences in flexibility might favor the adaptation of the RBD region or of the ACE2 surface by enabling a differently optimal interaction network. Interestingly, the complete flexibility profiles (Figure S6) suggest long-range effects of the mutations on the ACE2 and RBD dynamics, as most of the point mutations of the Alpha and Delta variants are not included in stiffer or more flexible regions compared to the WT system.

## 3. Materials and Methods

### 3.1. Molecular Dynamic Simulations

To characterize the impact of the point mutations in Alpha and Delta SARS-CoV-2 variants, we performed classical MD simulations using the NAMD 2.13 code (University of Illinois, Urbana–Champaign, IL, USA) [44]. The crystal structure of SARS-CoV-2 WT-RBD complexed with ACE2 has been extracted as a monomer from the Protein Data Bank (PDB) code 6M17. The structure of the Alpha variant was generated by adding the three mutations located in the RBD region (Figure 1) to the WT-RBD PDB. In particular, the procedure followed to generate mutated proteins was: (i) delete from the WT-RBD PDB the side chain atoms of the residue to be mutated, maintaining its backbone atoms, but associating them to the new residue; (ii) run the tleap Amber tool to automatically add the missing side chain atoms of the new residue.

Concerning the Delta-RBD variant, we used the available crystal structure of the complex formed with the ACE2 monomer (PDB code: 7W9I) and prepared it to fit with the crystal structure used for WT-RBD and Alpha-RBD variant. Each ACE2/RBD complex has been solvated with a cubic water box, described by a TIP3P water model [45,46]. The Amberff99SB force field [47] was selected to describe both proteins. To ensure the electroneutrality of the three built complexes (ACE2/WT-RBD, ACE2/Alpha-RBD, ACE2/Delta-RBD), K^+^ cations were added to neutralize the global negative charge of the systems. The Hydrogen Mass Repartition (HMR) algorithm [48] has been used to speed up the simulation time, scaling the mass of all non-water hydrogen atoms from 1.008 to 3.024 Da. This allows increasing the time step used to integrate Newton’s equations of motion from 2 fs to 4 fs, in combination with the RATTLE and SHAKE algorithms [49]. The molecular systems were first equilibrated in the constant pressure and temperature (NPT) ensemble, at a temperature of 300 K and a pressure of 1 atm, thanks to a Langevin thermostat and barostat [50], while including Periodic Boundary Conditions (PBC). Especially, the following protocol was used for every MD simulation: (i) 100,000 steps of minimization were performed to remove bad contacts; (ii) after, the systems were equilibrated during 36 ns progressively removing positional constraints on the proteins as follows: 12 ns with constraint scale 1.0, 12 ns with constraint scale 0.5, 12 ns with constraint scale 0.1; (iii) afterwards, MD production trajectories of 500 ns have been performed, doing three replicas for each system, hence accounting for a total simulation time of 1.5 µs per system (4.5 µs in total).

### 3.2. Computational Procedure for the Overall Structural Analysis

All results have been visualized and analyzed with the VMD software (version 1.9.3, University of Illinois, Urbana–Champaign, IL, USA) [51], the cpptraj program within the AmberTools22 (University of California, San Francisco, California, U.S.A.) [52], and BIOVIA Discovery Studio Visualizer (v20, Dassault Systèmes, Vélizy-Villacoublay, France) [53]. The analysis of RMSD and Solvent Accessible Surface Area (SASA) were performed using the VMD software. The RMSD was calculated using a plug-in tool implemented in VMD, while SASA was calculated through an analysis script publicly available at https://github.com/haileybureau/analysis_scripts/blob/master/sasa.tcl (accessed on 28 October 2022).

Hydrophilic and hydrophobic interactions were analyzed using the cpptraj program included in the AmberTools22. Hydrogen bonds’ analysis was performed to establish the hydrophilic interactions while distance analysis was performed to obtain the hydrophobic interactions. These analyses were performed considering the three MD runs of each system (a total of 37,500 frames). Concerning hydrophobic interactions, first we used BIOVIA Discovery Studio Visualizer, in particular the “receptor-ligand interaction” tool, to select the residues involved in the hydrophobic interactions. Then, distances between the center of mass of the residues’ side chains involved in the hydrophobic interaction were calculated with cpptraj. Finally, a distance criterion was applied to consider or not the presence of each hydrophobic interaction (interaction criteria: <6.00 Å for π-π T-shaped [54,55], <7.50 Å for π-π stacked [56,57], <6.0 Å for amide-π stacked [58,59], <6.5 Å for π-alkyl [54], and <4.0 Å for π-lone pair [60].

### 3.3. Clustering Analysis

Clustering analysis was performed using cpptraj within the AmberTools22, selecting the hierarchical agglomerative (bottom-up) algorithm. The choice of this algorithm follows the study of Shao et al. [61] recommending its use when the number of clusters is unknown, as it was the case here. Clustering analysis has been performed on 37,500 frames, that is, considering the three MD runs of each system, and has been applied on the RBD structure. The defined parameters are a cut-off of 3 Å between clusters and a maximum allowed of 10 clusters. “Average-linkage” has been used, which uses the average distance between members of two clusters. The most representative clusters for each run of each variant were analyzed.

### 3.4. Principal Component Analysis (PCA)-Based Flexibility Analysis

The flexibility analysis was performed using a script that was successfully applied to a variety of protein and nucleic acid models [62,63,64,65]. This script, inspired from the Scikit-learn58 library (KTH Royal Institute of Technology, Stockholm, Sweden) [66], allows to quantify the contribution of each residue to the dynamics of the system using a PCA-based machine learning protocol, within the kernel machines class of algorithms, that generates a covariance matrix based on the internal coordinates extracted from the output trajectory. The eigenmodes and eigenvectors of this matrix represent the modes of motion and the amplitude, respectively. Hence, eigenmodes with the highest amplitude correspond to the main fluctuations of the system, allowing to calculate the normalized contribution of each residue to these fluctuations. This analysis has been applied to both ACE2 and RBD, considering the three MD simulations performed for each system. The resulting flexibility (per residue) profiles are shown in Figure 5.

## 4. Conclusions

Through extended all-atom MD, we have compared the equilibrium behavior of the interface between the human ACE2 and the SARS-CoV-2 RBD, necessary to promote fusion of viral and cell membranes and therefore the infection of human body (Figure 1). In this study, ACE2 complexed to wild-type RBD, and to the two variants Alpha- and Delta-RBD were considered, to highlight structural differences at both global and residue-specific levels.

It was found that a global descriptor, previously applied to define the ACE2/RBD interaction strength (i.e., the distance between the centers of mass of the interacting proteins) [17,67], as well as a newly applied local descriptor (i.e., the flexibility indicator calculated by a kernel machine learning approach; see Figure 5) cannot fully describe the origin of the different interaction expected at the protein-protein interface, most probably due to the nature of the ACE2/RBD interface: a large and continuously changing area, that was fully depicted and discerned only when taking into account residue-by-residue hydrophilic and hydrophobic interactions (see Figure 2 and Figure 3). More in detail, also through a comparison with calculated SASA values (see Figure 4) and in agreement with previous experimental findings [26,27,28], it was clarified that the interaction strength between ACE2 and wild-type-RBD is similar when considering the Alpha variant, although lowering of the hydrophilic interactions is observed, due to a consistent increase of the hydrophobic interactions. The Delta variant, on the other hand, is expected to decrease its affinity for the ACE2, since hydrophilic interactions are in the same range as with wild-type RBD, but lacking almost every hydrophobic interaction.

In a more general term, with this work we highlight the importance to crosscheck different analysis tools of different nature (both methodologically-wise and physical chemistry-wise) in order to overcome pitfalls in assigning the relevance of specific bio-(macro)molecular interactions. Also, we would like to note that, within the quest for finding anti-COVID19 drugs, such protein-protein interactions are not limited to the ACE2/RBD interface, since other highly relevant targets were studied by this and other groups during these pandemics’ years. Among targets, the most common were the proteases Mpro (or 3CLpro) and PLpro, but also the SARS Unique Domain (SUD) and the RNA-dependent RNA polymerase [67,68,69,70,71,72,73,74].

## Figures and Tables

**Table 1:** Average RMSD values and distance between the mass centers of ACE2 and RBD.

	RMSD			ACE2/RBD Mass Centers Distance
	ACE2/RBD Complex	ACE2	RBD	
WT	3.4 Å	2.9 Å	3.4 Å	47.70 Å
Alpha	3.8 Å	3.5 Å	2.9 Å	47.07 Å
Delta	4.2 Å	3.2 Å	2.6 Å	47.14 Å

## Data Availability

Data is contained within the article or supplementary material.

## Supplementary Materials

The following supporting information can be downloaded at: https://www.mdpi.com/article/10.3390/ijms24032517/s1. Figure S1: Time series of the RMSD of the ACE2/RBD complex; Figure S2: Time series of the RMSD of the ACE2 protein; Figure S3: Time series of the RMSD of the RBD protein; Figure S4: Time series of the distance between the center of mass of ACE2 and RBD; Figure S5: Time series of the SASA analysis on the ACE2/RBD complex; Figure S6: Complete flexibility profiles for RBD and ACE2; Table S1: Hydrophilic interaction values at the ACE2/RBD interface; Table S2: Hydrophobic interaction values at the ACE2/RBD interface; Table S3: Hydrophilic and hydrophobic interaction values at the ACE2/RBD interface detailed for each cluster.
