# Supplementary material for: Revealing the Molecular Interactions between Human ACE2 and the Receptor Binding Domain of the SARS-CoV-2 Wild-Type, Alpha and Delta Variants
Source: Int J Mol Sci. 2023 Jan 28;24(3):2517. doi: 10.3390/ijms24032517 (PMC9916449; doi:10.3390/ijms24032517)
Supplement: Supplementary file 1 [file ijms-24-02517-s001.zip › ijms-2164486-supplementary.pdf]

Supporting information

Revealing the molecular interactions between human ACE2 and the receptor binding domain of the SARS-CoV-2 wild-type, Alpha and Delta variants

Cécilia Hognon,[a] Emmanuelle Bignon,[b] Antonio Monari,[c] Marco Marazzi,[a,d]*Cristina Garcia-Iriepa,[a,d]*

[a]Universidad de Alcalá, Departamento de Química Analítica, Química Física e Ingeniería Química, Ctra. Madrid-Barcelona, Km 33,600, 28871, Alcalá de Henares, Madrid, Spain.
[b]Université de Lorraine and CNRS, UMR 7019 LPCT, F-5400 Nancy, France.
[c]Université de Paris and CNRS, ITODYS, F-75006 Paris, France
[d]Universidad de Alcalá, Instituto de Investigación Química “Andrés M. del Río” (IQAR), 28871 Alcalá de Henares, Madrid, Spain

*cristina.garciai@uah.es, marco.marazzi@uah.es

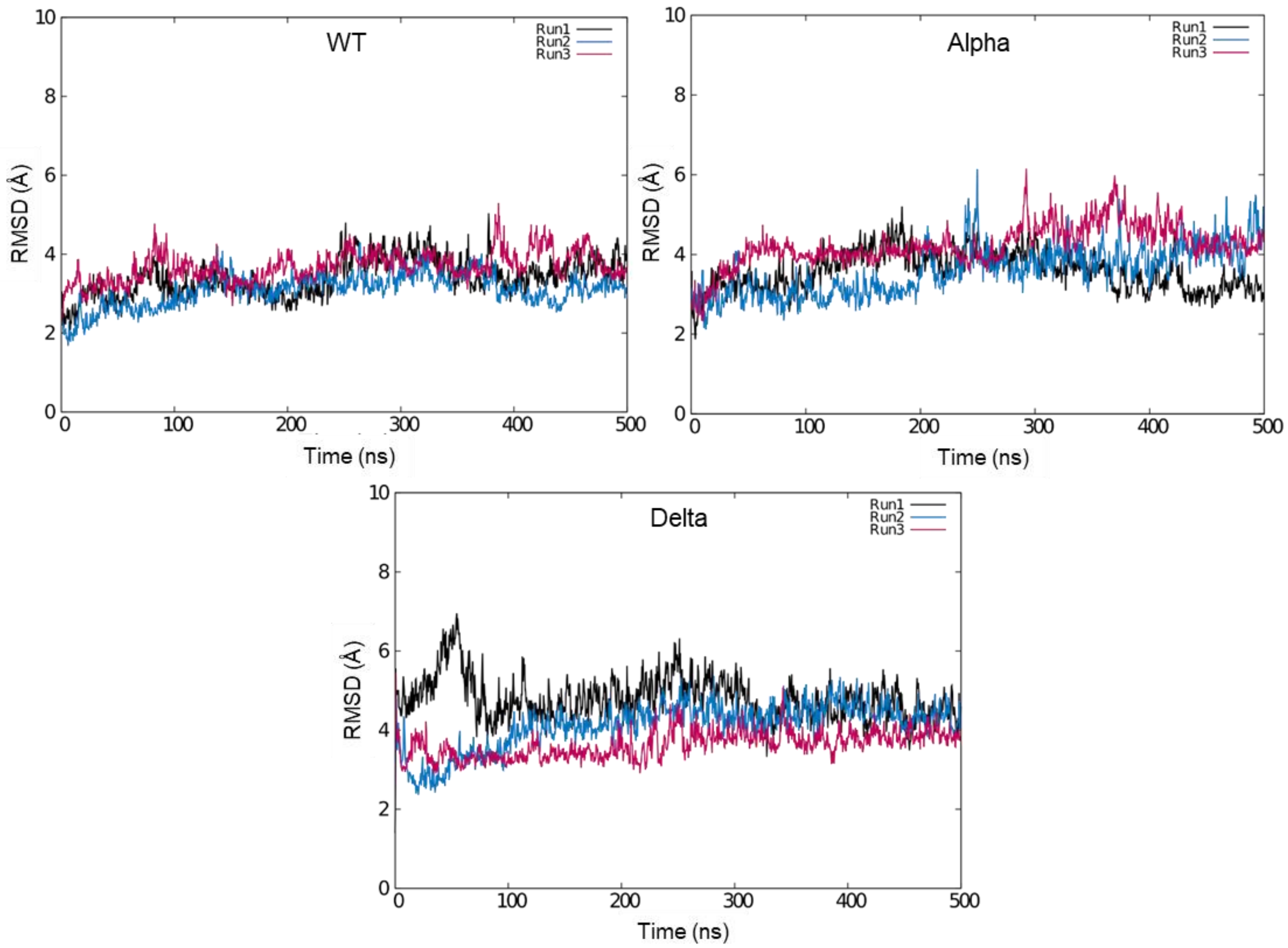

Figure S1: Time series of the RMSD analysis for each variant of the ACE2/RBD complex. The three runs of each variant are represented.

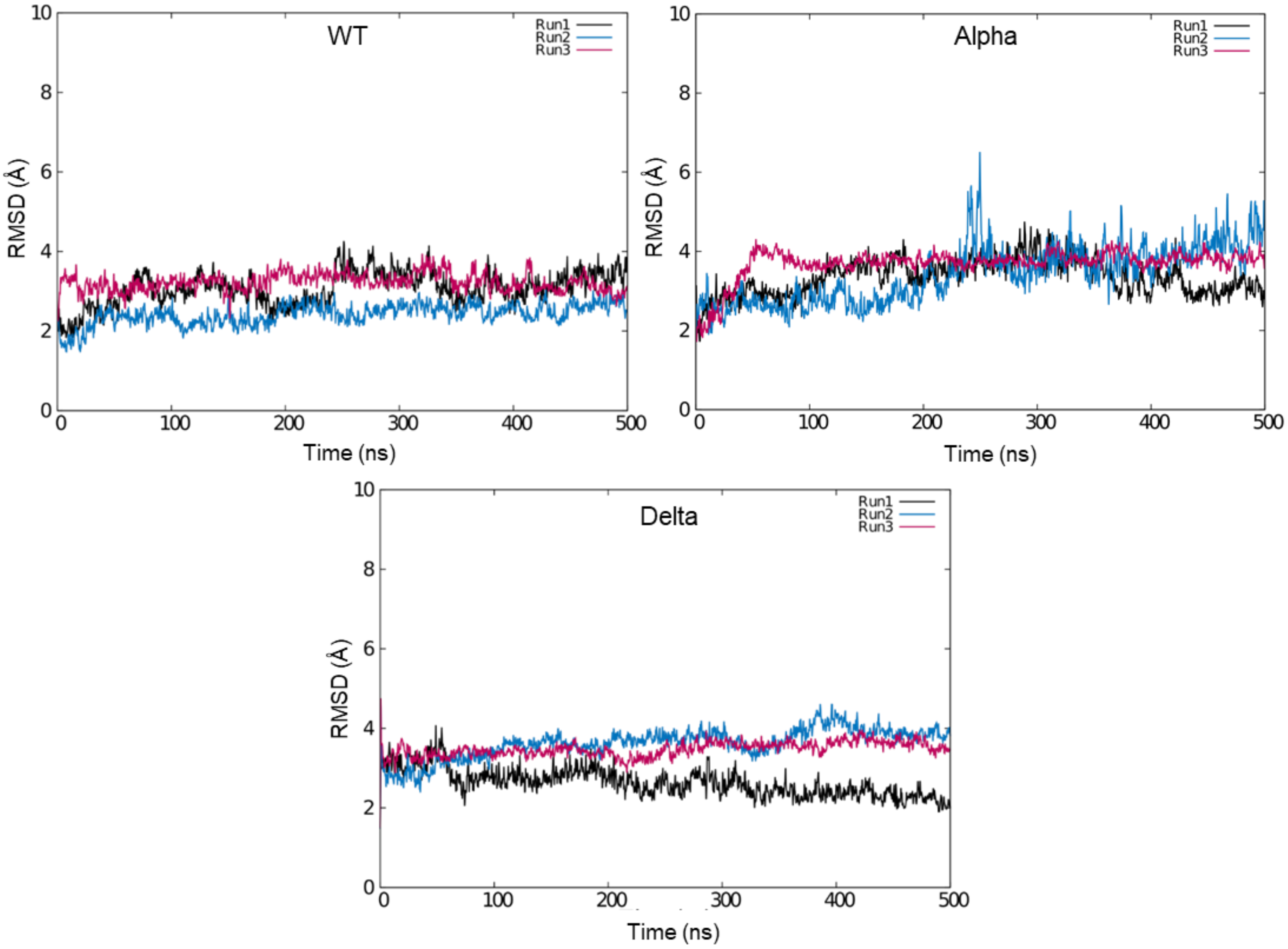

Figure S2: Time series of the RMSD analysis for each variant of the ACE2 protein. The three runs of each variant are represented.

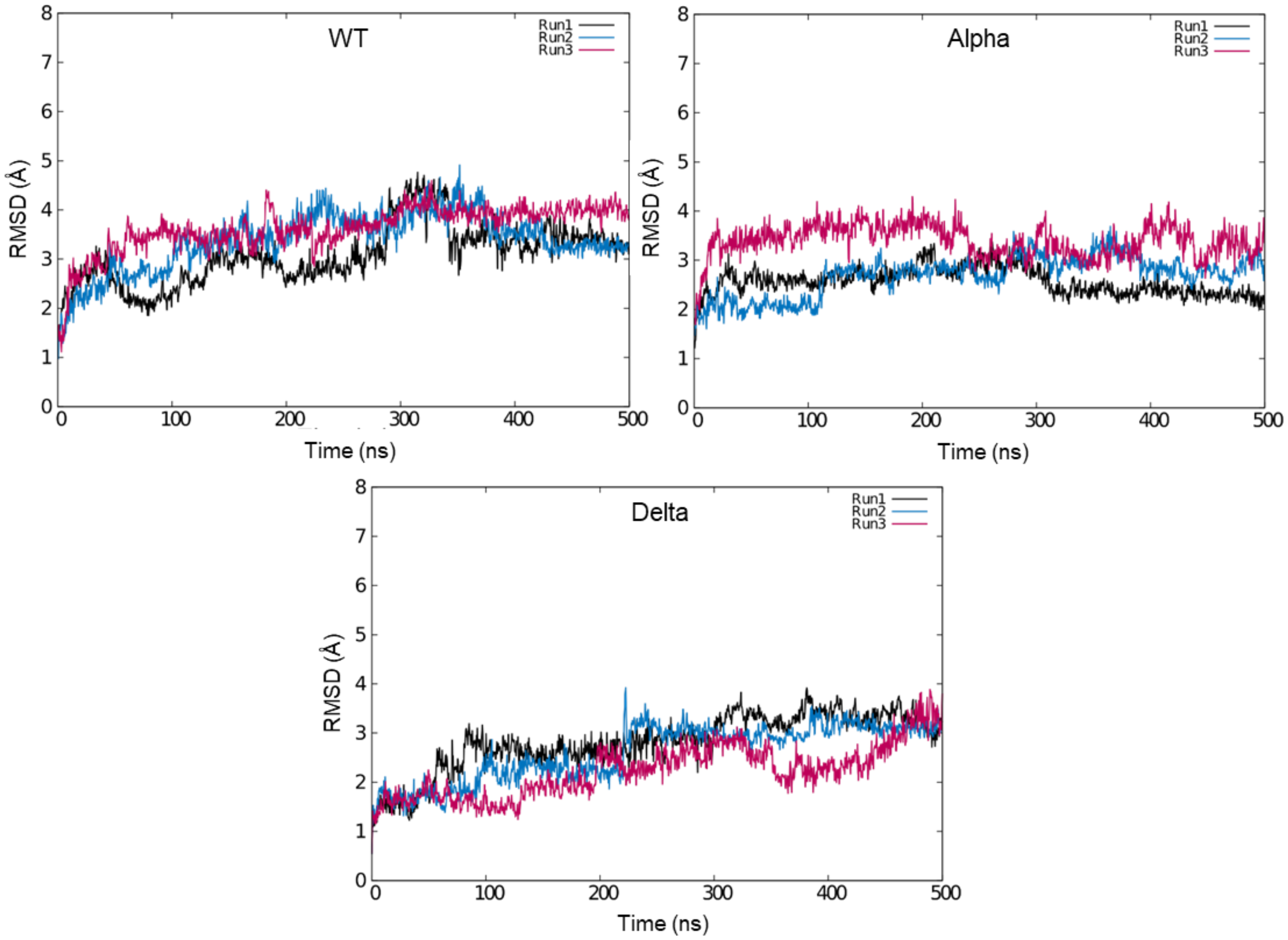

Figure S3: Time series of the RMSD analysis for each variant for the RBD protein. The three runs of each variant are represented.

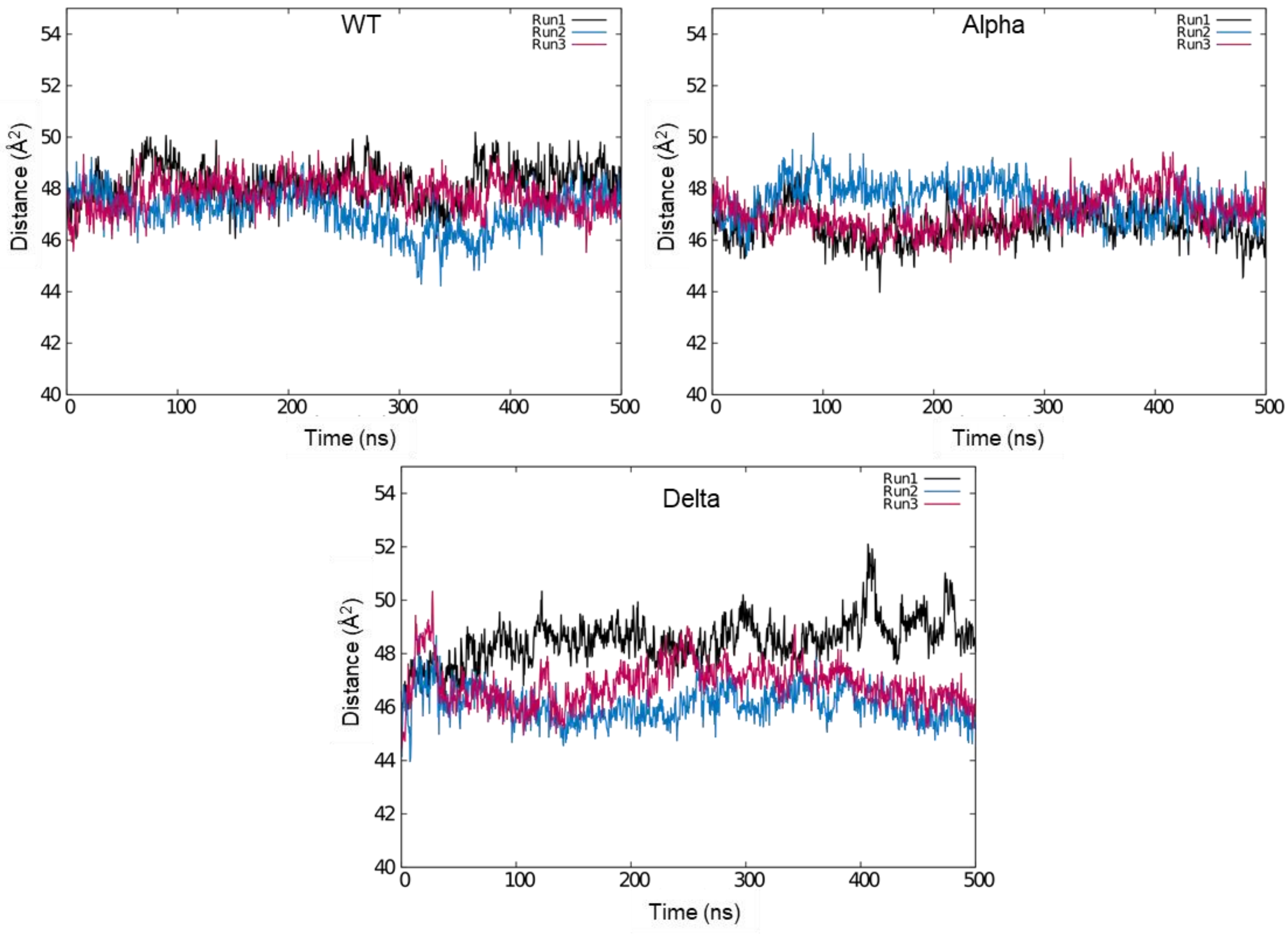

Figure S4: Time series of the distance between the centers of mass of ACE2 and RBD for each variant. The three runs of each variant are represented.

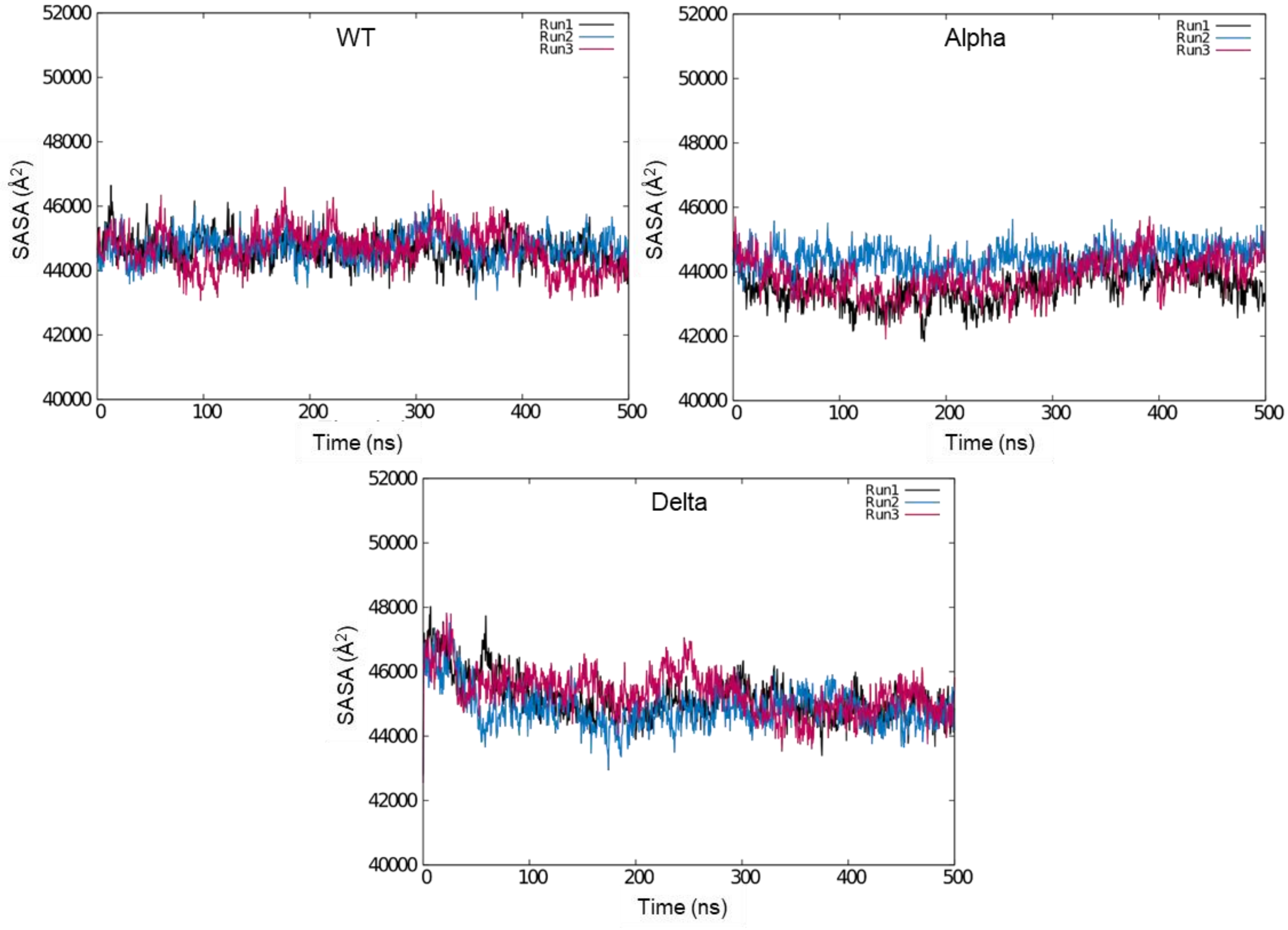

Figure S5: Time series of the SASA analysis for each variant applied on the ACE2/RBD complex. The three runs of each variant are represented.

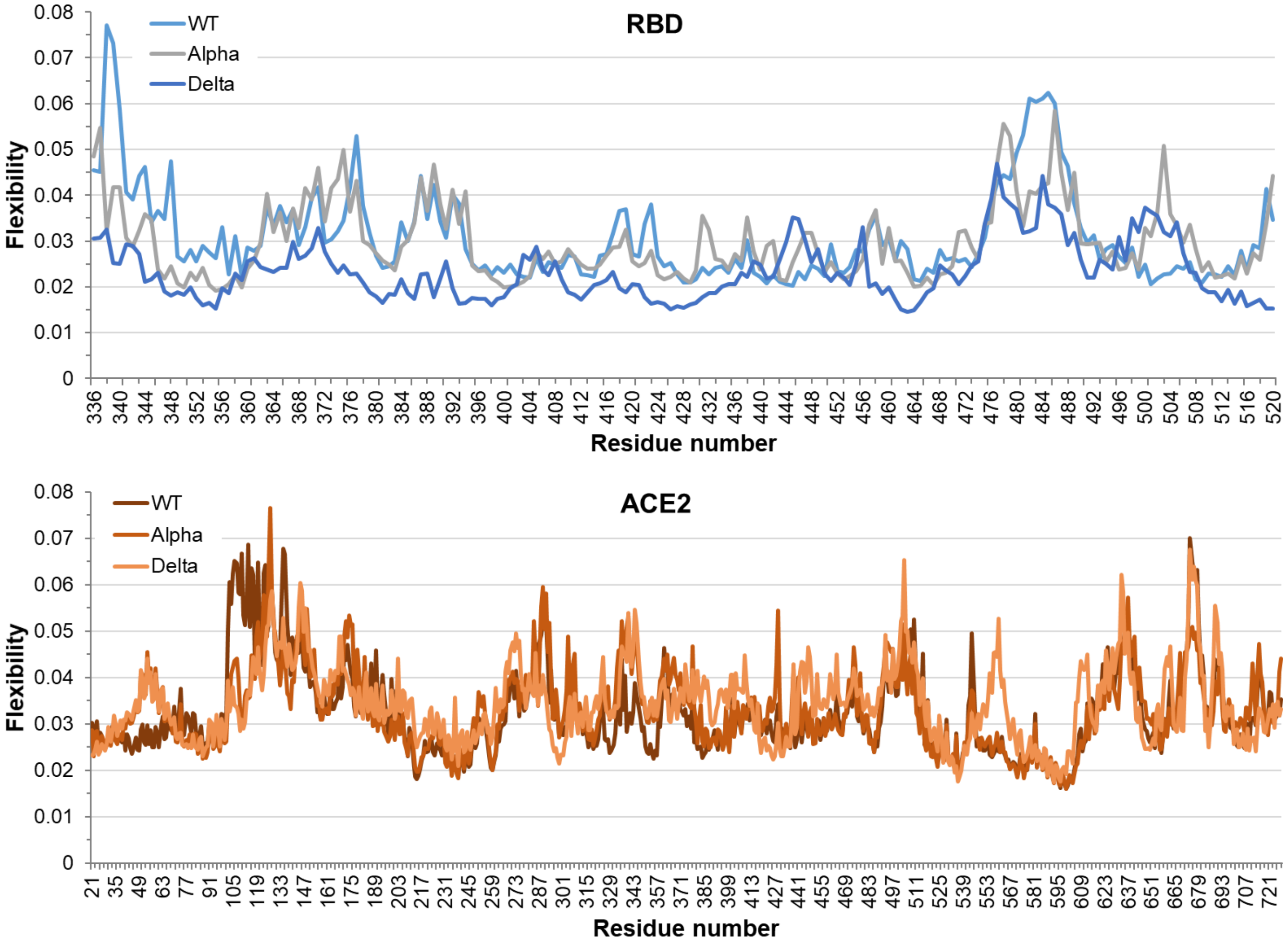

Figure S6: Flexibility profiles of all residues of RBD (top) and ACE2 (bottom) computed by machine learning post-analysis, for the WT and the different variants.

Table S1: Hydrophilic interaction values at the ACE2/RBD interface for WT, Alpha, and Delta strain. Point mutations are depicted in red.

WT

RBD amino acid	ACE2 amino acid	Average Fraction
LYS 417	ASP 30	0.475
TYR 421	ASP 30	0.033
GLY 446	GLN 42	0.049
TYR 449	ASP 38	0.683
TYR 453	HIS 34	0.116
TYR 473	THR 27	0.011
GLN 474	THR 27	0.047
GLN 474	GLU 23	0.016
GLN 474	GLN 24	0.024
ALA 475	GLN 24	0.170
SER 477	GLN 24	0.021
SER 477	GLU 23	0.057
THR 478	GLN 24	0.110
THR 478	GLU 23	0.036
GLU 484	LYS 31	0.142
ASN 487	TYR 83	0.185
ASN 487	GLN 24	0.118
TYR 489	TYR 83	0.031
GLN 493	GLU 35	0.447
GLN 493	HIS 34	0.078
GLN 493	LYS 31	0.117
SER 494	HIS 34	0.299
TYR 495	LYS 353	0.022
GLY 496	LYS 353	0.159
GLN 498	LYS 353	0.193
GLN 498	GLN 42	0.046
GLN 498	ASP 38	0.036
THR 500	ASP 355	0.765
THR 500	TYR 41	0.096
THR 500	ASN 330	0.012
ASN 501	LYS 353	0.016
GLY 502	LYS 353	0.684
TYR 505	GLU 37	0.553
TYR 505	ARG 393	0.021

Alpha

RBD amino acid	ACE2 amino acid	Average Fraction
LYS 417	ASP 30	0.534
LYS 439	GLU 329	0.103
LYS 439	GLN 325	0.033
TYR 449	ASP 38	0.831
GLN 474	GLN 24	0.057
ALA 475	GLN 24	0.172
GLY 476	TYR 83	0.035
GLY 476	GLN 24	0.038
SER 477	MET 82	0.105
SER 477	GLU 23	0.033
THR 478	GLN 24	0.095
GLU 484	LYS 31	0.113
ASN 487	TYR 83	0.190
ASN 487	GLN 24	0.028
ASN 487	GLU 75	0.045
ASN 487	GLN 76	0.015
TYR 489	THR 27	0.040
GLN 493	GLU 35	0.465
GLN 493	LYS 31	0.062
SER 494	HIS 34	0.407
GLN 498	LYS 353	0.056
GLN 498	TYR 41	0.022
THR 500	ASP 355	0.415
THR 500	TYR 41	0.151
THR 500	ASN 330	0.050
THR 500	LYS 353	0.194
THR 500	GLY 354	0.045
THR 500	GLY 326	0.037
TYR 501	GLU 37	0.112
TYR 501	MET 383	0.015
GLY 502	LYS 353	0.405
TYR 505	GLU 37	0.184
TYR 505	ALA 386	0.019
GLN 506	GLN 325	0.023

Delta

RBD amino acid	ACE2 amino acid	Average Fraction
LYS 417	ASP 30	0.659
ASN 439	GLN 25	0.128
GLY 446	GLN 42	0.024
GLY 446	LYS 353	0.025
TYR 449	ASP 38	0.580
TYR 449	GLN 42	0.011
TYR 453	HIS 34	0.068
TYR 473	GLN 24	0.020
ALA 475	SER 19	0.101
ALA 475	GLN 24	0.065
GLY 476	GLN 24	0.032
SER 477	GLN 24	0.014
SER 477	GLN 76	0.043
ASN 487	TYR 83	0.431
ASN 487	GLN 24	0.184
TYR 489	TYR 83	0.081
GLN 493	GLU 35	0.380
GLN 493	HIS 34	0.072
GLN 493	LYS 31	0.119
SER 494	HIS 34	0.193
GLY 496	LYS 353	0.107
GLN 498	LYS 353	0.264
GLN 498	GLY 354	0.010
GLN 498	ASP 38	0.024
GLN 498	ASP 355	0.039
THR 500	ASP 355	0.498
THR 500	TYR 41	0.108
THR 500	GLN 325	0.073
THR 500	GLY 354	0.235
THR 500	THR 324	0.167
GLY 502	LYS 353	0.483
VAL 503	THR 324	0.029
TYR 505	GLU 37	0.283
TYR 505	ARG 393	0.013
TYR 505	ALA 386	0.039
GLN 506	GLN 325	0.016

Table S2: Hydrophobic interaction values at the RBD/ACE2 interface for WT, Alpha and Delta strain. Point mutations are depicted in red.

RBD amino acid	ACE2 amino acid	Type	Average Fraction
**WT**			
PHE 453	HIS 34	π-π T-shaped	0.46
PHE 486	LEU 79	π Alkyl	0.38
PHE 486	MET 82	π Alkyl	0.72
PHE 486	TYR 83	π-π T-shaped / π-π stacked	0.99
TYR 489	LYS 31	π Alkyl	0.88
TYR 505	LYS 353	π Alkyl	0.99
**Alpha**			
PHE 453	HIS 34	π-π T-shaped	0.75
PHE 456	LYS 31	π Alkyl	0.37
PHE 486	MET 82	π Alkyl	0.81
PHE 486	TYR 83	π-π T-shaped	0.56
TYR 489	LYS 31	π Alkyl	0.98
TYR 501	TYR 41	π-π T-shaped	0.94
TYR 501	LYS 353	π Alkyl	1.00
TYR 505	LYS 353	π Alkyl	1.00
TYR 505	LYS 353	Amide π stacked	1.00
**Delta**			
TYR 453	HIS 34	π lone pair / π-π T-shaped	0.11
PHE 486	MET 82	π Alkyl	0.28
PHE 486	TYR 83	π-π stacked	0.25
TYR 505	LYS 353	Amide π stacked	0.08
TYR 505	LYS 353	π Alkyl	0.06
TYR 505	ALA 387	π Alkyl	0.85

Table S3: Hydrophilic and hydrophobic interaction details for each cluster for WT, Alpha and Delta strain. Point mutations are depicted in red.

WT

Cluster 0		Cluster 1		Cluster 2	
**RBD amino acid**	**ACE2 amino acid**	**RBD amino acid**	**ACE2 amino acid**	**RBD amino acid**	**ACE2 amino acid**
**Hydrophilic interaction**					
LYS 417	ASP 30	THR 500	ASP 355	GLY 446	GLN 42
TYR 449	ASP 38	TYR 505	GLU 37	TYR 449	ASP 38
ALA 475	GLN 24			SER 494	HIS 34
GLN 493	GLU 35			GLN 498	LYS 353
	LYS 31			THR 500	ASP 355
SER 494	HIS 34			ASN 501	LYS 353
THR 500	ASP 355			GLY 502	LYS 353
GLY 502	LYS 353			TYR 505	GLU 37
TYR 505	GLU 37				
**Hydrophobic interaction**					
PHE 486	TYR 83	PHE 486	TYR 83	TYR 453	HIS 34
TYR 489	LYS 31	PHE 486	MET 82	PHE 486	LEU 79
TYR 505	LYS 353	TYR 489	LYS 31	TYR 489	LYS 31
		TYR 505	LYS 353	TYR 505	LYS 353

Alpha

Cluster 0 (Run1)		Cluster 1 (Run3)		Cluster 2 (Run2)	
**RBD amino acid**	**ACE2 amino acid**	**RBD amino acid**	**ACE2 amino acid**	**RBD amino acid**	**ACE2 amino acid**
**Hydrophilic interaction**					
LYS 417	ASP 30	LYS 417	ASP 30	LYS 417	ASP 30
LYS 439	GLU 329	TYR 449	ASP 38	LYS 439	GLU 329
TYR 449	ASP 38	ALA 475	GLN 24	TYR 449	ASP 38
GLN 493	GLU 35	ASN 487	TYR 83	GLY 476	TYR 83
SER 494	HIS 34	SER 494	HIS 34	GLN 493	GLU 35
THR 500	ASP 355			SER 494	HIS 34
TYR 505	GLU 37			THR 500	ASN 330
	ARG 393			GLY 502	LYS 353
**Hydrophobic interaction**					
PHE 453	HIS 34	PHE 453	HIS 34	PHE 453	HIS 34
PHE 456	LYS 31	PHE 456	LYS 31	TYR 489	LYS 31
PHE 486	MET 82	PHE 486	MET 82	TYR 501	LYS 353
PHE 486	TYR 83	TYR 489	LYS 31	TYR 501	TYR 41
TYR 489	LYS 31			TYR 505	LYS 353
TYR 501	LYS 353			TYR 505	LYS 353
TYR 501	TYR 41				
TYR 505	LYS 353				

Delta					
Cluster 0 (Run3)		Cluster 1 (Run2)		Cluster 2 (Run1)	
**RBD amino acid**	**ACE2 amino acid**	**RBD amino acid**	**ACE2 amino acid**	**RBD amino acid**	**ACE2 amino acid**
**Hydrophilic interaction**					
LYS 417	ASP 30	TYR 449	ASP 38	LYS 417	ASP 30
TYR 449	ASP 38	ASN 487	TYR 83	ASN 439	GLN 325
ALA 475	SER 19	TYR 489	TYR 83	ASN 487	GLN 24
ASN 487	TYR 83	GLN 493	GLU 53	GLN 498	ASP 355
THR 500	ASP 355	THR 500	ASP 355	THR 500	GLY 354
TYR 505	GLU 37	TYR 505	GLU 37		
**Hydrophobic interaction**					
PHE 486	MET 82	TYR 453	HIS 34	TYR 505	ALA 387
PHE 486	TYR 83	PHE 486	MET 82		
TYR 505	ALA 387	PHE 486	TYR 83		
TYR 505	LYS 353	TYR 505	LYS 353		
TYR 505	LYS 353	TYR 505	LYS 353		
